# Trophic Selective Pressures Organize the Composition of Endolithic Microbial Communities From Global Deserts

**DOI:** 10.3389/fmicb.2019.02952

**Published:** 2020-01-08

**Authors:** Evan B. Qu, Chris R. Omelon, Aharon Oren, Victoria Meslier, Don A. Cowan, Gillian Maggs-Kölling, Jocelyne DiRuggiero

**Affiliations:** ^1^Department of Biology, Johns Hopkins University, Baltimore, MD, United States; ^2^Department of Geography and Planning, Queen’s University, Kingston, ON, Canada; ^3^Institute of Life Sciences, The Hebrew University of Jerusalem, Jerusalem, Israel; ^4^Centre for Microbial Ecology and Genomics, Department of Biochemistry, Genetics and Microbiology, University of Pretoria, Pretoria, South Africa; ^5^Gobabeb Namib Research Institute, Walvis Bay, Namibia

**Keywords:** endolithic, desert, xerotolerant, biogeography, trophic level, microbial assembly

## Abstract

Studies of microbial biogeography are often convoluted by extremely high diversity and differences in microenvironmental factors such as pH and nutrient availability. Desert endolithic (inside rock) communities are relatively simple ecosystems that can serve as a tractable model for investigating long-range biogeographic effects on microbial communities. We conducted a comprehensive survey of endolithic sandstones using high-throughput marker gene sequencing to characterize global patterns of diversity in endolithic microbial communities. We also tested a range of abiotic variables in order to investigate the factors that drive community assembly at various trophic levels. Macroclimate was found to be the primary driver of endolithic community composition, with the most striking difference witnessed between hot and polar deserts. This difference was largely attributable to the specialization of prokaryotic and eukaryotic primary producers to different climate conditions. On a regional scale, microclimate and properties of the rock substrate were found to influence community assembly, although to a lesser degree than global hot versus polar conditions. We found new evidence that the factors driving endolithic community assembly differ between trophic levels. While phototrophic taxa, mostly oxygenic photosynthesizers, were rigorously selected for among different sites, heterotrophic taxa were more cosmopolitan, suggesting that stochasticity plays a larger role in heterotroph assembly. This study is the first to uncover the global drivers of desert endolithic diversity using high-throughput sequencing. We demonstrate that phototrophs and heterotrophs in the endolithic community assemble under different stochastic and deterministic influences, emphasizing the need for studies of microorganisms in context of their functional niche in the community.

## Introduction

Arid and hyper-arid regions of Earth pose a significant challenge to life, with stresses such as high solar radiation, sparse and intermittent rain events, and drastic temperature fluctuations exerting strong selective pressures on organisms (Potts and Webb, 1994; Lebre et al., 2017). As aridity increases in an area and the diversity of higher eukaryotic life decreases, microorganisms begin to constitute a larger component of the total biomass and provide essential ecosystem functions such as carbon fixation and biogeochemical cycling (Pointing and Belnap, 2012; Makhalanyane et al., 2015). Desert microorganisms colonize rock and soil matrix as an adaptation to the harsh environmental conditions, forming a category of microbial refuges that includes biological soil crusts, hypoliths (under rock), epiliths (above rock), and endoliths (within rock) (Pointing and Belnap, 2012).

Endolithic communities, which inhabit pore and fissure space within the interior of rocks, are perhaps the most xerotolerant of these microbial refuges, appearing in the driest environments on Earth such as the hyper-arid core of the Chilean Atacama Desert and the McMurdo Dry Valleys in Antarctica (Friedmann and Ocampo, 1976; Azua-Bustos et al., 2012). These communities usually colonize a thin layer underneath the surface of the rock and have been described in diverse substrates including sandstone (Friedmann et al., 1967; Walker and Pace, 2007), limestone (Wong et al., 2010), halite (de los Ríos et al., 2010), gypsum (DiRuggiero et al., 2013), ignimbrite (Wierzchos et al., 2013), and granite (de los Ríos et al., 2005). Depending on the nature of the substrate, the primary mode of community colonization can either be cryptoendolithic, inhabiting pores within the rock matrix or chasmoendolithic, inhabiting cracks and fissure space in the rocks (Golubic et al., 1981). These communities are characterized by low taxonomic and trophic diversity and are dominated by a few core photoautotrophic taxa along with a variety of chemoheterotrophic taxa (Wierzchos et al., 2018). *Cyanobacteria* are typically the dominant phototroph, often belonging to the multi-resistant genus *Chroococcidiopsis* (Friedmann et al., 1967; Meslier et al., 2018; Wierzchos et al., 2018), although eukaryotic green algae have also been reported in sandstone (Walker and Pace, 2007), gypsum (Wierzchos et al., 2015) and halite communities from the Atacama Desert (Robinson et al., 2015). Diverse heterotrophic bacteria are found in the endolithic community, with the phyla *Actinobacteria*, *Proteobacteria*, *Chloroflexi, Bacteroidetes*, and *Deinococcus-Thermus* particularly represented (Walker and Pace, 2007; Meslier et al., 2018; Wierzchos et al., 2018).

Desert lithic habitats have been well-utilized for studies of microbial biogeography, as their low diversity, simple trophic structure, and similar microenvironment provides a tractable model when investigating spatial patterns in microbial community assembly (Caruso et al., 2011). These studies have found that environmental determinants, in particular climate, are the primary factors that organize microbial communities at a global and regional scale (Friedmann, 1980; Chan et al., 2012; Lacap-Bugler et al., 2017). Biotic interactions are also thought to influence community assembly, and hypolithic *Cyanobacteria* have been reported to play a key role in determining overall community structure and function (Valverde et al., 2015). Conversely, it remains poorly understood how stochastic processes influence assembly within the lithic habitat. Early studies of endolithic sandstones found remarkably similar communities at global scales, which led to the hypothesis that these communities are seeded from a global metacommunity (Walker and Pace, 2007). However, more recent work of hypolithic communities have found support for the existence of dispersal limitation between deserts (Bahl et al., 2011; Caruso et al., 2011; Archer et al., 2019). Much of the work investigating lithic habitats over spatial scales has been done using the quartz hypolithic system (Pointing et al., 2007, 2009; Warren-Rhodes et al., 2007a, b) and only a few global surveys of the endolithic system exist (Friedmann, 1980; Walker and Pace, 2007). While hypolithic communities colonize the underside of quartz rocks at the interface with the underlying soil, endolithic communities colonize a diversity of rock substrates, have been found to be distinct from hypolithic communities within the same local environment (Van Goethem et al., 2016), and can even colonize more extreme environments (Warren-Rhodes et al., 2007a), raising the question of whether the same processes govern the assembly of both lithic habitats.

The central question this study seeks to answer is what factors determine the assembly of global desert endolithic communities. A variety of environmental effects have been proposed to influence endolithic community assembly, including macroclimate (Friedmann, 1980), rock geochemistry (Walker and Pace, 2007), and substrate architecture (Meslier et al., 2018), but study limitations and differing methodologies have made it difficult to place the relative importance of these factors. It also remains untested as to whether stochastic processes play a role in controlling endolithic community assembly. To investigate this question, we conducted a comprehensive survey of endolithic communities and relevant abiotic factors at global, continental, and regional scales. In order to focus on macroscopic influences, we only sampled sandstones to limit effects from the rock microenvironment, which have been shown to strongly affect community composition even within the same local environment (Crits-Christoph et al., 2016; Meslier et al., 2018).

## Materials and Methods

### Sample Collection and DNA Extraction

**FIGURE 1:**
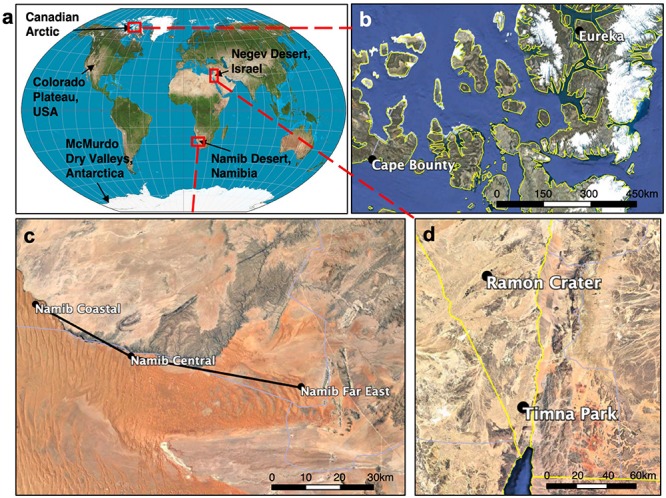
Map of global and regional sandstone sampling sites. **(a)** World map showing sampling locations. In the Canadian Arctic **(b)**, Namib Desert **(c)**, and Negev Desert **(d)** samples were taken from multiple sites whose names and locations are shown. More detailed information can be found in Supplementary Table S1.

Colonized endolithic sandstones were collected from five desert regions (Negev Desert, Namib Desert, Colorado Plateau, Canadian Arctic, McMurdo Dry Valleys) during the summer months between 2013 and 2018 (Supplementary Table S1). In the Namib Desert, Negev Desert, and Canadian Arctic, samples were collected from multiple regional sites (Figure 1). Rock samples were broken off with a field hammer and stored in sterile WhirlPak bags (Nasco) in the dark at room temperature, except for samples from the Canadian Arctic and McMurdo Dry Valleys, which were stored in the dark at −20°C.

DNA was extracted from ten separate rocks for each of the nine desert sites, for a total of 90 samples overall. Extraction was done with 0.25 ± 0.05 g of rock scraped from colonized areas with a sterile metal tool using the PowerSoil DNA Isolation Kit (Qiagen). With the exception of Antarctic and Negev samples, DNA extraction was conducted within 3 months of collection. For Antarctica and Timna Park, DNA was extracted in two occasions a year apart; this did not have a significant impact on community composition (ANOSIM; *p* = 0.409 and *p* = 0.366, respectively), indicating that storage conditions did not significantly influence community composition. To validate the robustness of the sequencing pipeline, duplicate DNA extraction libraries were constructed for two samples at each site and sequenced separately. Post-extraction, DNA was quantitated with the Qubit Fluorometer dsDNA HS Assay (Thermo Fisher Scientific). DNA extraction negative controls did not return bands on a gel or signal in Bioanalyzer DNA High-Sensitivity (Agilent) traces and were not sequenced.

### Climate Data

For each site, temperature, relative humidity, precipitation, and solar irradiance were obtained from meteorological sources for a 2-year period encompassing the dates during which samples were collected (Supplementary Table S2). Daily solar radiation was found to be very consistent within desert sampled, and so a single average value was reported for each desert. In the Negev Desert, rainfall data were taken from 2017 to 2018, due to abnormal rainfall events in 2016 after sample collection that increased the 2-year average. For the University Valley site, solar radiation data were obtained from a study of the nearby Beacon Valley.

### Scanning Electron Microscopy

Small colonized rock fragments were broken off from sandstones and fixed in 3.0% formaldehyde, 1.5% glutaraldehyde, 5 mM Ca^2+^, 2.5% sucrose, in 0.1M sodium cacodylate, pH 7.4 at room temperature for 30 min. Afterward, samples were washed thrice in 0.1M sodium cacodylate, 10 min each. Samples were stained with 1% OsO_4_ in 0.1M sodium cacodylate in the dark for 45 min, followed by three quick washes in ddH_2_O. Stained samples were dehydrated in a cold ethanol gradient: 50, 75, 90, 95%, then three 15 min changes in 100% ethanol at room temperature, followed by a final dehydration in hexamethyldisilazane for 2 min. Finally, samples were coated with a 1.5 nm Pt layer before imaging in a FEI Quanta 200 Environmental SEM. Imaging was done at 15 kV, spot size 1.5, 6 mm working distance for high-magnification imaging and 20 kV, spot size 2.5, 10 mm working distance for low-magnification imaging.

### Measurement of Physical and Chemical Sandstone Properties

For each site, a combined 15 g of crushed, non-colonized sandstone from three separate rocks was analyzed for mineral composition through borate fusion X-ray fluorescence, performed at SGS Canada Inc. (Lakefield, Canada). Sandstone samples were tested for soluble NO_3_^–^, PO_4_^3–^, Cl^–^, and SO_4_^2–^ via ion chromatography, EPA method 300.0 and soluble Ca^2+^, Mg^2+^, K^+^, Na^+^, Fe^2+^, and Fe^3+^ via inductively coupled plasma atomic emission spectroscopy, EPA method 200.7. Soluble ion analyses were performed at Inter-Mountain Labs (Sheridan, WY, United States).

Grain size distribution of sandstone samples was obtained through sieve analysis (Krumbein and Pettijohn, 1938). Sandstones were first disaggregated by gently crushing 25 g of rock with a pestle and mortar, taking care not to pulverize individual sandstone grains. Rock samples were shaken through a series of sieves with opening sizes 710, 500, 355, 250, 180, 125, 90, 60 μm for 10 min, after which mass in each sieve was measured. Cumulative grain size distributions were fitted to a sigmoid function, from which a 50th percentile diameter (D50) value was extracted for each rock. Water retention of sandstone rocks was measured through a fluid resaturation method. Rock samples in triplicate were first fully dried for 24 h in an oven, then weighed. Samples were then immersed in distilled water for 3 days to allow the rock to fully saturate, after which wet mass and bulk volume (V_B_) were measured. Pore volume (V_P_) was obtained by subtracting dry mass from wet mass and multiplying by the density of water, and a percent water retention was calculated using the formula 100(*V*_*P*_−*V*_*B*_) (U. S. Department of the Interior and U. S. Geological Survey, 1963).

### 16S rRNA Library Generation and Sequencing

Libraries for 16S rRNA gene and ITS amplicons were created using a two-step PCR protocol. The 16S rRNA gene was first amplified using primer pair 515F–GTGYCAGCMGCCGCGG TAA and 806R–GGACTACNVGGGTWTCTAAT targeting the V3-V4 hypervariable region (Caporaso et al., 2012). For ITS amplicon libraries, primer pair fits7F–GTGARTCA TCGAATCTTTG and ITS4R – TCCTCCGCTTATTGATATGC was used (Ihrmark et al., 2012). The primer pairs ITS1F/ITS4R (Gardes and Bruns, 1993) and ITS86F/ITS4R (Turenne et al., 1999) were also tested on all samples to ensure eukaryotic diversity was thoroughly detected, but these did not lead to any additional amplification.

PCR reactions were constructed using: 4–40 ng of template DNA, 1X Phusion PCR HF Buffer, 0.5 mM MgCl_2_ 3% DMSO, 800 μM dNTP mix, 0.25 μM of each primer, 0.02 U/μL of Phusion High-Fidelity DNA Polymerase (New England Biolabs), and nuclease-free water for a total volume of 25 μL/reaction, and run with the following cycling conditions: 98°C for 30 s, followed by 20 cycles of 98°C for 10 s, 55°C for 15 s, 72°C for 15 s, final extension at 72°C for 5min. First step PCR reactions were done in duplicate for each sample, then pooled. After each PCR step, products were cleaned with a 0.6X Sera-Mag Speedbeads mixture (Roland and Reich, 2012), then quantitated with the Qubit Fluorometer dsDNA HS Assay (Thermo Fisher Scientific) before further downstream processing.

Barcode and adapter sequences were attached in a second step PCR reaction as follows: 4 ng of cleaned first step PCR product, 1X Phusion PCR HF Buffer, 0.5 mM MgCl_2_ 3% DMSO, 800 μM dNTP mix, 0.36 μM universal forward primer, 0.36 μM uniquely barcoded reverse primer, 0.02 U/μL of Phusion High-Fidelity DNA Polymerase (New England Biolabs), and nuclease-free water were combined for a total volume of 25 μL/reaction. Cycling conditions for second step reactions were 98°C for 30 s, followed by 10 cycles of 98°C for 10 s, 55°C for 15 s, 72°C for 15 s, final extension at 72°C for 5 min. Equimolar amounts of each library were pooled before sequencing, and final multiplexed libraries were quality-checked using the 2100 Bioanalyzer DNA High-Sensitivity Kit (Agilent) before sequencing. Two nM libraries were sequenced on the Illumina 2500 MiSeq platform at read length 2 × 250 bp in the Genetic Resources Core Facility at Johns Hopkins University. Sequence data were demultiplexed and non-biological sequences were removed prior to analysis.

### Analysis and Visualization of Sequence Data

Eighty-nine 16S rRNA gene libraries and 29 ITS2 libraries from sandstone microbial communities were successfully sequenced, along with control replicates used to validate the robustness of the sequencing pipeline (Supplementary Table S1). Paired-end sequencing at 250 bp returned 7,700,178 paired 16S rRNA gene reads and 2,070,702 paired ITS reads. Both bacterial and eukaryotic sequencing depths sampled community diversity to asymptote (Supplementary Figure S1).

The bioinformatics package QIIME2, ver. 2018.11 was used to de-noise Illumina reads (Bolyen et al., 2018). Forward and reverse reads were manually trimmed at the 3′ end once the lower quartile of sequences dropped below a Phred score of 20. Quality filtering, dechimerization, and inference of exact sequence variants (ESVs) was done with the ‘dada2’ plugin in QIIME2 (Callahan et al., 2016). Sequencing runs were independently processed in the DADA2 pipeline and then consolidated. Due to high stochastic sequence variation at the ESV level, 16S rRNA gene analysis was performed after clustering ESVs into operational taxonomic units (OTUs). Using the ‘vsearch’ plugin in QIIME2 (Rognes et al., 2016) and the SILVA 16S rRNA reference database, release 128 (Quast et al., 2013), bacterial ESVs were clustered into open-reference OTUs at 97% similarity, returning 3227 OTUs and a 2.8-fold decrease in bins. ITS sequence analysis was done entirely at the ESV level due to the difficulties in accurately clustering ITS sequence data by identity (Popovic et al., 2018).

Taxonomy was assigned to eukaryotic ESVs and bacterial OTUs using a naïve-Bayes classification approach from the ‘q2-feature-classifier’ package in QIIME2 (Bokulich et al., 2018). The classifier was trained on the SILVA 16S rRNA reference database, release 128 at 97% identity (Quast et al., 2013) for bacterial sequences and the UNITE database ver. 7.2 at 97–99% dynamic identity for eukaryotic sequences (Nilsson et al., 2019). ITS taxonomy was supplemented by performing BLASTn alignment of unassigned sequences against the NCBI nr/nt database and assigning consensus taxonomy from the top 10 hits. Mantel, adonis, and ANOSIM statistical tests, as well as Pearson correlation coefficient were calculated in QIIME2. Taxonomy, heatmap and PCoA visualizations were constructed in R using the ggplot2 and phyloseq packages. To generate bipartite networks, OTUs were first normalized using relative abundance of reads, then node associations were calculated with the make_otu_network.py function in QIIME, release 1.9.1 (Caporaso et al., 2010). Network visualization was performed in Cytoscape, ver. 3.7.1 (Shannon et al., 2003).

## Results

**FIGURE 2:**
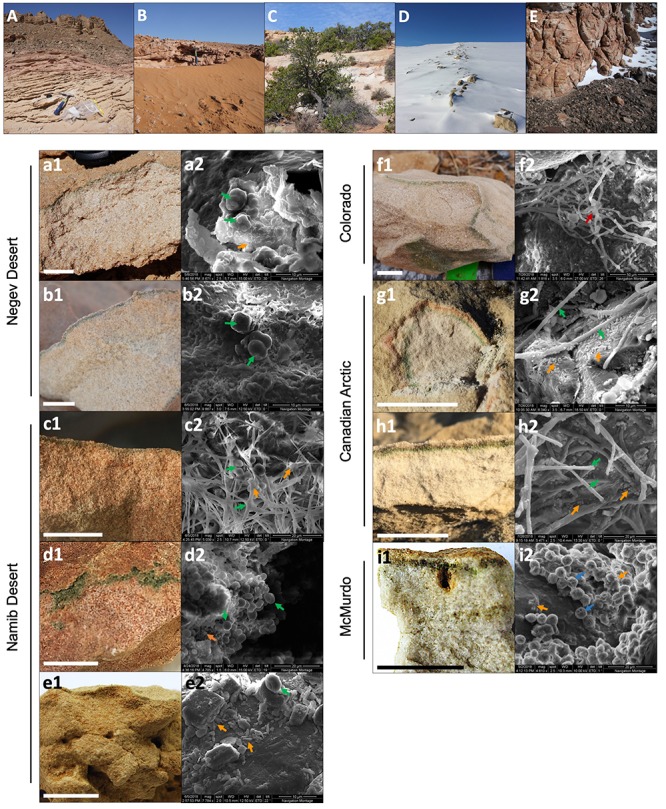
Location, colonization zone, and SEM micrographs of microbial communities from each sampling site. **(A–E)** General views of the five deserts sampled: **(A)** Negev Desert, **(B)** Namib Desert, **(C)** Colorado Plateau, **(D)** Canadian Arctic, and **(E)** McMurdo Dry Valleys. **(a1–i1)** Sandstone colonization zone at each site, in the order: **(a)** Timna Park, **(b)** Ramon Crater, **(c)** Namib Coastal, **(d)** Namib Central, **(e)** Namib Far East, **(f)** Escalante, **(g)** Eureka, **(h)** Cape Bounty, **(i)** University Valley. Scale bar = 2 cm. **(a2–i2)** SEM micrograph images of the endolithic microbial communities. Arrows point to morphologies consistent with cyanobacterial forms (green), fungal hyphae (red), green algae (blue), and heterotrophic bacteria-like cells (orange).

Colonized endolithic sandstones were sampled in five deserts and nine sites (Figure 1). Among deserts, a wide range of environmental conditions were observed (Figures 2A–E and Supplementary Table S2). We grouped these deserts into three distinct climate regimes based on mean temperature, solar irradiance, and moisture variables (Supplementary Table S2). Hot deserts (Namib and Negev Deserts) had high solar irradiance and air temperatures reaching up to 40°C in the summer months, as well as lower relative humidity. Polar deserts (Antarctic Dry Valleys and Canadian Arctic) were characterized by long winters with complete darkness and very low temperatures, followed by short summers with above-zero temperatures and near-constant sunlight. Temperate deserts (Colorado Plateau) was primarily characterized by high precipitation and relative humidity, along with moderate temperatures.

### Characterization of Endolithic Colonization Zones

In all sandstones, a green colonization zone was observed a few millimeters below the surface of the rock (Figures 2a–i). The colonization was mostly cryptoendolithic with the exception of sandstone from central Namib, where colonization was found in small crevices parallel to the rock surface (chasmoendolithic; Figure 2d). In Arctic and Antarctic sandstones, an additional orange (Figure 2g) or brown (Figures 2h,i) pigmented layer was located above the green colonization layer.

SEM imaging revealed microbial cells in dense aggregates within the colonized zone. Large coccoidal and filamentous cells consistent with *Cyanobacteria* (Figures 2a–h) and green algae morphologies (Figure 2i) were the most prominent feature within the microbial landscape. These morphologies varied between deserts and sites, ranging from mostly coccoidal in the Negev Desert (Figures 2a,b) to filamentous in the Canadian Arctic (Figures 2g,h). Coccoidal cyanobacteria-like cells had a diameter of 3–4 μm, while filamentous cyanobacteria-like cells were around 1 μm in diameter and up to 50 μm long. Large algae-like cells, 5–6 μm in diameter, were observed only in the Antarctic University Valley community. Coccoidal and bacilliform cells with morphologies consistent with heterotrophic bacteria were also found in all communities; their sizes ranged between 0.5 and 1 μm in the longest dimension. Filamentous shapes consistent with fungal hyphae morphologies measuring up to 20 μm wide and over 200 μm long were observed in the Colorado Plateau community (Figure 2f). Microbial cells were often found surrounded by extracellular polymeric substances (EPS), with the organization of EPS varying greatly between deserts and sites. For example, in Negev Desert samples, cells were organized in clusters and encased entirely in EPS (Figures 2a,b).

### Rock Architecture and Chemical Properties

X-ray fluorescence (XRF) analysis showed that SiO_2_ was the most abundant compound in all sandstones, followed by Fe_2_O_3_ and Al_2_O_3_. The compounds CaO, MgO, K_2_O, Na_2_O, TiO_2_, and MnO were present in all sandstones at abundances less than 2%, while P_2_O_5_, Cr_2_O_3_, and V_2_O_5_ were sparsely present in sandstones at abundances less than 0.1% (Supplementary Table S3). Namib Central sandstones had a unique XRF profile with high percentages of CaO (15.8%) and loss on ignition mass (13.5%), suggesting an enrichment in calcium carbonate, which breaks down into CaO and O_2_ during analysis (Supplementary Table S3). Water-soluble ions remained at concentrations below 30 mg/kg. Soluble Fe^2+^, Fe^3+^, and PO_4_^3–^ were below detection limits (Supplementary Table S4). Sandstone grain size was variable between sites, with the D50 of sandstone grains ranging from 116 to 530 μm (Supplementary Table S5 and Supplementary Figure S2). Water retention capacity ranged from 10.9 to 28.0% v/v (Supplementary Table S5).

### Endolithic Community Composition

**FIGURE 3:**
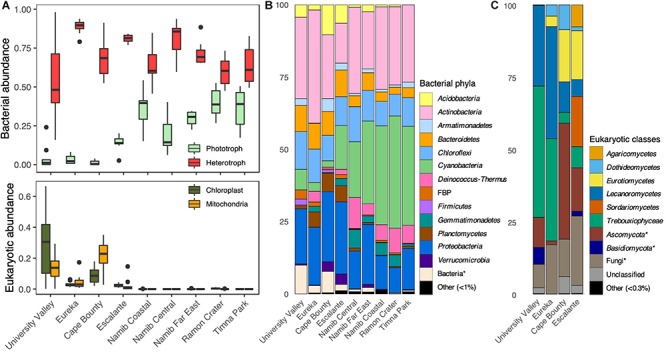
Sandstone endolithic community composition. **(A)** Top: Relative proportions of bacterial phototrophs (oxygenic and anoxygenic phototrophs) and heterotrophs, obtained from 16S rRNA gene sequences Bottom: Relative proportions of chloroplast and mitochondrial 16S rRNA gene sequences. **(B)** Prokaryotic community composition, based on 16S rRNA gene sequences. **(C)** Eukaryotic community composition based on ITS sequences. Asterisks indicate a lack of taxonomic information up to the designated level.

High-throughput sequencing of the 16S rRNA gene and ITS regions was used to characterize sandstone endolithic communities at the molecular level. Bacteria and Eukarya were the primary members of the endolithic community, while Archaea were only present at very low relative proportions (<0.1%) in every desert except the McMurdo Dry Valleys (Figure 3A). The most predominant bacterial phyla were *Cyanobacteria*, *Actinobacteria*, and *Proteobacteria* (∼95% *Alphaproteobacteria*), which together represented 48–75% of the communities (Figure 3B). The taxonomic assignment of fungal ITS sequences performed particularly poorly, with 44% of ITS sequence variants only assignable to the phylum level or higher. However, the data showed that the eukaryotic community was comprised of fungi belonging to the phyla *Ascomycota* (60 ± 26%) and *Basidiomycota* (4 ± 14%) and green algae belonging to the *Trebouxiophyceae* (20 ± 23%) and *Chlorophyceae* (<0.1%) (Figure 3C). Archaea primarily belonged to the phylum *Thaumarchaeota*. Mitochondrial (mt) and chloroplast (cp) DNA reads in the 16S rRNA gene sequencing data were used as a rough proxy for the relative presence of eukaryotes within the overall microbial community (Figure 3A). mtDNA and cpDNA were only detected in the polar deserts (University Valley, Eureka, Cape Bounty) and to a lower extent, in the temperate Escalante community, matching the sites for which ITS amplicons could be obtained (Figure 3C). Among the polar desert sites, University Valley and Cape Bounty had a higher presence of eukaryotes (Figure 3A), although this data should be approached with caution due to the high variability of mitochondria and chloroplast counts among eukaryotes.

Photosynthesizing *Cyanobacteria* and green algae constituted the highest proportion of reads in the community and were dominated by the genera *Chroococcidiopsis* and *Trebouxia*, respectively (Figures 3B,C). There was a significant negative relationship between the relative proportions of *Cyanobacteria* and algae reads (Pearson; ρ = −0.539, *p* < 0.001). This was especially true for the University Valley and Eureka sites, where *Trebouxia* dominated and few *Cyanobacteria* were detected (Figures 3B,C). A small portion (0.4–3%) of 16S rRNA genes reads belonged to clades with anoxygenic phototrophic members, including *Acetobacteraceae*, *Rhodospirillaceae*, *Rhodobacteraceae*, *Bradyrhizobiaceae*, *Comamonadaceae*, and *Gemmatimonas* (Supplementary Table S7); however, further genus-level annotation revealed several chemoheterotrophic taxa among these clades. The low relative abundance of anoxygenic phototrophic reads indicate that this type of metabolism likely constituted a minor component of our community.

### Global Effects of Endolithic Community Assembly

**FIGURE 4:**
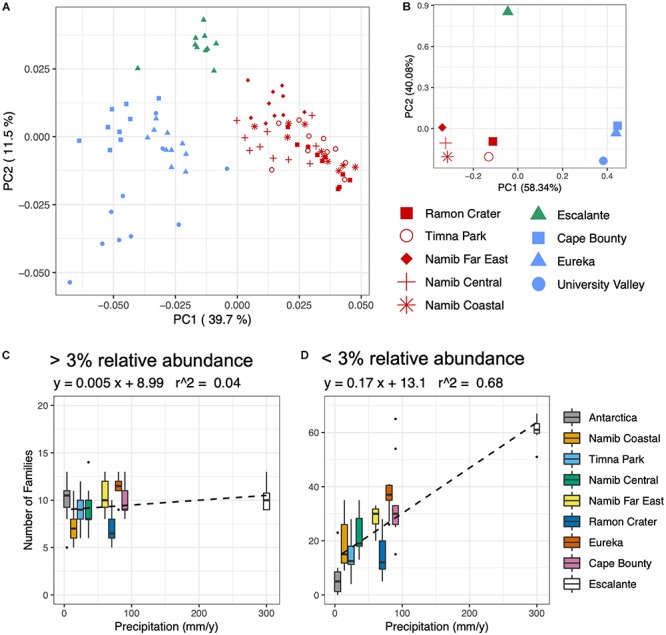
Environmental correlations with bacterial community composition and diversity. **(A)** Principal coordinates analysis (PCoA) plot of weighted UniFrac distance of the prokaryotic community, based on 16S rRNA gene sequences rarefied to 1500 reads. Sites are colored according to climate regime, with red = hot/dry, blue = polar, and green = temperate. **(B)** PCA of Euclidean distance of climate variables: average temperature, precipitation, relative humidity, daily solar radiation. **(C,D)** Boxplots of number of families plotted against yearly precipitation for **(C)** families comprising over 3% of reads and **(D)** families comprising below 3% of reads.

Initial patterns of bacterial community assembly were analyzed through principal coordinates analysis (PCoA) of weighted UniFrac distance (Figure 4A). Communities significantly clustered according to their respective climate regime (ANOSIM; *R* = 0.87, *p* < 0.001); this sample grouping alone explained 46% percent of the variation between communities (adonis; *R* = 0.46, *p* < 0.001). Among individual climate variables, average air temperature was the most correlated with community distance, followed by yearly solar irradiance and distance between sampling sites (Figure 4B and Table 1). Only a few substrate variables were significantly correlated with community distance: soluble K^+^, percent Fe_2_O_3_, and percent Al_2_O_3_ (Table 1).

**TABLE 1:** Significant correlations (*p* < 0.01) of abiotic variables with prokaryotic alpha and beta diversity using Pearson and Mantel tests.

**Beta diversity (Mantel)**	**Predictor**	**Rho**
	Air Temperature	0.725
	Solar Irradiance	0.587
	Geographic Distance	0.585
	Relative Humidity	0.384
	Soluble K^+^	0.308
	Precipitation	0.279
	Fe_2_O_3_	0.189
	Al_2_O_3_	0.179

**Alpha Diversity (Pearson)**	**Predictor**	**Rho**

	Precipitation	0.885
	Fe_2_O_3_	–0.607
	Relative Humidity	0.569
	Grain Size	–0.530

Precipitation was the variable most correlated with bacterial and eukaryotic alpha diversity; grain size, relative humidity and percent Fe_2_O_3_ were also significantly correlated (Table 1). The community response to precipitation was remarkably different for highly and lowly represented taxa, with the strongest effect evident at the family level (Figures 4C,D). The endolithic community harbored 7–13 highly abundant families (>3% of total reads) independently of moisture conditions (*R*^2^ = 0.04), but the number of lowly abundant families (<3% of total reads) increased in a positive linear relationship (*R*^2^ = 0.68) from around 5 families at the driest site (University Valley) to over 60 at the wettest (Escalante). Sites with higher moisture regimes often saw the appearance of new taxonomic groups, such as *Oscillatoriales* among the *Cyanobacteria*, *Chlorophyta* among the green algae, and *Sordariomycetes* and *Agaricomycetes* among the fungi. Anoxygenic phototrophic bacteria were also associated with higher moisture regimes, being present in Cape Bounty, Eureka, Escalante, and the Far East and Central Namib Desert, but absent in all other sites (Supplementary Table S7).

### Fine-Scale Partitioning of the Sandstone Microbial Community

**FIGURE 5:**
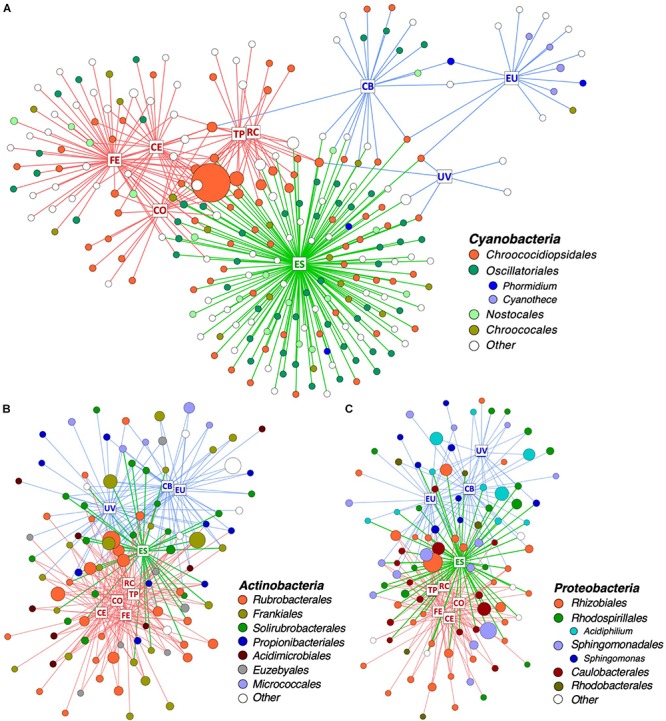
Distribution of OTUs across desert sites. Bipartite network of **(A)** all *Cyanobacteria* and the 100 **(B)** *Actinobacteria* and **(C)** *Proteobacteria* OTUs with highest proportion of reads. Squares represent sites and are labeled with site name: RC, Ramon Crater; TP, Timna Park; CO, Namib Coastal; CE, Namib Central; FE, Namib Far East; ES, Escalante; CB, Cape Bounty; EU, Eureka; UV, University Valley. Edges are colored in according to the climate regime of their respective site: red = hot, blue = polar, and green = temperate. Circles represent OTUs and are colored by taxonomic order and genus. Singleton taxa and unassigned OTUs are colored in white. The size of each circle is proportional to mean relative proportion of that OTU.

In order to identify which taxa drive community differences between climate regimes, bipartite networks were generated for the three most dominant bacterial phyla: *Cyanobacteria*, *Actinobacteria*, and *Proteobacteria* (Figure 5). Network analysis revealed that *Cyanobacteria* operational taxonomic units (OTUs) were particularly specific to a single climate regime, with only 18% of cyanobacterial OTUs shared between two or more climate regimes (Figure 5A and Supplementary Figure S3). Hot deserts (Figure 5A, red boxes) had a cyanobacterial profile that was dominated by *Chroococcidiopsis*, with a single *Chroococcidiopsis* OTU (accession number FJ790616) constituting a high proportion of reads in all hot desert communities. Polar deserts (Figure 5A, blue boxes) had the lowest cyanobacterial diversity among the climate regimes, especially at University Valley (UV) and Eureka (EU). *Chroococcidiopsis*, *Cyanothece*, and *Phormidium* were the most common *Cyanobacteria* found in polar deserts. The highest cyanobacterial diversity was located in the temperate Escalante (ES) site (Figure 5A, green letters in boxes), which uniquely harbored 47% of cyanobacterial OTUs and had higher taxonomic diversity than other deserts. The wetter Namib Far East (FE) site also had higher OTU and taxonomic diversity, albeit to a lesser extent.

Non-cyanobacterial OTUs were distributed in a more cosmopolitan manner, with 54% of *Actinobacteria* OTUs (Figure 5B and Supplementary Figure S3) and 65% of Proteobacteria OTUs (Figure 5C and Supplementary Figure S3) shared between two or more climate regimes. OTU richness among *Proteobacteria* and *Actinobacteria* did not vary between climate regimes, but some differences in taxonomic distribution were noticed between regimes. For instance, 34% of *Actinobacteria* OTUs in hot deserts belonged to the single genus *Rubrobacter* and the proteobacterial genera *Acidiphilum* and *Sphingomonas* were mostly specific to polar deserts.

### Regional Impacts on Endolithic Community Assembly

**FIGURE 6:**
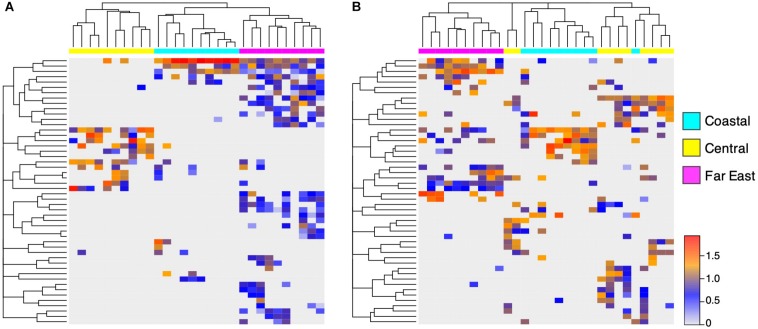
Regional differences in Namib Desert communities. UPGMA-clustered heatmap of 50 **(A)** *Cyanobacteria* OTUs and **(B)** *Proteobacteria* OTUs with highest proportion of reads, from three sites in the Namib Desert. Rows correspond to OTUs and columns correspond to individual samples. Color scale represents log-normalized relative abundances.

Sandstones were collected at multiple sites within the Canadian Arctic, Namib Desert, and Negev Desert to further investigate within-desert impacts on community assembly (Figure 1). Significant community differences were observed between regional sites in the Canadian Arctic (ANOSIM; *R* = 0.70, *p* < 0.001) and Namib Desert (ANOSIM; *R* = 0.81, *p* < 0.001), but not the Negev Desert (ANOSIM; *R* = 0.14, *p* = 0.06). In the Canadian Arctic, the eukaryotic community at Eureka was low-diversity, containing 29 ± 10 exact sequence variants (ESVs) and dominated by *Trebouxia* (36%), while the eukaryotic community at Cape Bounty was high-diversity, containing 74 ± 14 ESVs, while *Trebouxia* had low relative proportions (4% of total reads) (Figure 1 and Supplementary Table S6). Within the Namib Desert, hierarchical clustering analysis at the OTU level showed that bacterial profiles generally clustered according to site, but this grouping was much more rigorous for *Cyanobacteria* than for *Actinobacteria* and *Proteobacteria* (Figure 6 and Supplementary Figure S4). Notably, the Namib Far East site harbored a much higher cyanobacterial diversity than the other two Namib sites, but not a considerably higher diversity of other bacteria.

## Discussion

We sampled endolithic sandstones across three major biogeographic regions (North America, Africa/Asia, and Antarctica) and three diverse climate regimes in order to investigate long-range patterns of diversity in endolithic communities. Previous global studies of the endolithic system only reported samples from two regions, using low-resolution molecular analysis (Friedmann, 1980; Walker and Pace, 2007). Our sandstone samples had mostly similar geochemical properties, thus confirming that the sandstone endolithic system is a consistent model for studying macroscopic patterns in microbial ecology.

The bacterial composition of the sandstone community was consistent with previous findings from sandstones (Walker and Pace, 2007; Goordial et al., 2016; Archer et al., 2017), as well as other endolithic substrates (DiRuggiero et al., 2013; Khomutovska et al., 2017; Meslier et al., 2018). The ubiquitous phyla in nearly all endolithic substrates were *Cyanobacteria*, *Alphaproteobacteria*, and *Actinobacteria*, while the phyla *Acidobacteria*, *Deinococcus-Thermus*, *Bacteroidetes*, *Chloroflexi*, and *Planctomycetes* were commonly found but at lower relative abundances. The eukaryotic component of the endolithic community has been more poorly characterized, and the sparse taxonomic annotation of ITS sequences in this study suggests that much of the eukaryotic diversity within endoliths remains to be discovered. Despite this, our findings concur with previous studies which report that the endolithic eukaryotic community is solely comprised of fungi from the phyla *Ascomycota* and *Basidiomycota* along with a low diversity of *Trebouxiophyceae* primarily belonging to the genus *Trebouxia* (Pointing et al., 2009; Archer et al., 2017). Archaea have been poorly reported in endolithic communities to the point where their presence is questioned (Pointing et al., 2009; Meslier et al., 2018); however, we found archaeal signatures in every site except University Valley, indicating that Archaea do contribute to the sandstone endolithic community, albeit at very low relative abundances. Our findings using molecular data support a number of previous studies which characterize sandstone endolithic communities using microscopic methods (Friedmann, 1980; Bell et al., 1986; Omelon et al., 2006).

We did not find support for geographic distance alone being a primary driver of community assembly, and it is likely that the biogeographic signal in our data is mostly an artifact of environmental differences between continents. The most convincing evidence for this is the remarkable similarity in community composition between the Arctic and Antarctic communities and the bipolar distribution of many taxa, a finding which has been replicated in soil (Cox et al., 2016) and biofilm communities (Kleinteich et al., 2017). Highly similar communities were also found between the hot deserts; these results together suggest that environmental filtering, rather than dispersal limitation, is the primary organizing principle in endolithic communities. While previous studies have used similar findings as evidence for global dispersal of microorganisms throughout the atmosphere (Walker and Pace, 2007), a preponderance of recent evidence has been found in support of widespread dispersal limitation among microbial communities (Moeller et al., 2017; Power et al., 2018; Archer et al., 2019). In Antarctica, the high aerial environment has been shown to be particularly selective and limits dispersal of many microbial taxa to and from the continent (Archer et al., 2019). This raises the possibility that the bipolar distribution of certain OTUs may be a remnant of ancient biogeography (Bahl et al., 2011).

We pinpoint macroclimate as the primary driver of global endolithic community assembly. The greatest difference in community composition was observed between polar and hot desert conditions. The hot desert communities were almost entirely bacterial and dominated by *Cyanobacteria*. In contrast, communities from polar deserts had greatly reduced relative abundances of *Cyanobacteria* and saw an increased presence of fungi and green algae. The temperate Colorado Plateau community supported rich cyanobacterial and eukaryotic populations and was the most diverse site by far. This difference in community composition between hot and cold deserts has been well-studied by Friedmann (1980), who first posited that conditions in hot deserts were actually harsher for life than in polar deserts. In hot deserts, high daytime evapotranspiration causes the endolithic habitat to be desiccated for a majority of the time, with only a short period of time in the early morning suitable for photosynthesis (Friedmann, 1980). Conversely, in polar deserts, humidity and constant solar radiation during summer months creates a relatively warm and wet environment in the rock matrix in spite of below-freezing outside conditions (Omelon et al., 2006). Thus, the patterns of global community composition are likely a result of eukaryotic life being mostly excluded from the harsher hot deserts. Analyses of endolithic communities from cold, low-latitude desert environments such as the Tibetan Plateau and Eastern Pamir mountains find similar community profiles, with lowered cyanobacterial proportions and increased presence of eukaryotes, thus supporting the idea that cold deserts are less extreme than hot deserts (Wong et al., 2010; Khomutovska et al., 2017).

Moisture availability has generally been assumed to be the most important factor for desert communities under constant xeric stress (Pointing et al., 2007; Lebre et al., 2017; Neilson et al., 2017); however, we found that moisture variables such as precipitation and relative humidity are secondary drivers of global community assembly, behind hot versus polar conditions. The most noticeable community response to higher moisture was an increase in the number of rare taxa with low proportions of reads, covering many additional taxonomic groups. These rare taxa were often desert-specific, supporting the concept that stochastic assembly gains increased importance at higher moisture regimes (Chase, 2010; Lee et al., 2018). On the other hand, the composition and diversity of dominant and presumably well-adapted taxa were found to be remarkably similar across moisture regimes, particularly at the family taxonomic level. This is in contrast with desert soil communities, where major reorganization from drier to wetter conditions has been reported (Neilson et al., 2017; Lee et al., 2018; Scola et al., 2018). The consistency in diversity and taxonomy across various moisture regimes suggests that a core community exists with a functional landscape that is well-adapted to the sandstone endolithic habitat, regardless of moisture regime. We should note that this observation is not necessarily true when comparing rock substrates of different compositions and physico-chemical properties, as significant differences were observed in the composition and diversity of communities from different types of substrates found side-by-side in the Atacama Desert (Meslier et al., 2018). We also found evidence for increased metabolic diversity with higher moisture, as several anoxygenic phototrophic clades were present at low proportions only in wetter environments. However, these clades contain many representatives with multiple metabolisms, and so the true contribution of anoxygenic phototrophs may be smaller than the percentages indicate.

Sandstone rocks have been found to support cryptoendolithic communities (Walker and Pace, 2007); however, we also found a unique chasmoendolithic community in the calcium carbonate-rich Namib Central sandstone, supporting a cyanobacterial profile not found in other Namib sandstones. Both the chasmoendolithic and cryptoendolithic Namib sandstone were still dominated by the genus *Chroococcidiopsis*, but they harbored substrate-specific OTUs, suggesting specialization of closely related species depending on substrate properties. Fine-scale partitioning of microbial diversity by substrate type has been described in calcite, ignimbrite and gypsum rocks and mainly attributed to “rock architecture,” meaning the space availability and water retention properties of the rock matrix (Crits-Christoph et al., 2016; Meslier et al., 2018). Other properties of the rock substrate, such as geochemical composition and soluble ion availability were generally not found to influence microbial assembly, with the exception of Fe_2_O_3_ and Al_2_O_3_ abundances. While these minerals might directly influence community physiology, they might also impact community assembly by affecting light transmission properties of the rock (McKay, 2012).

We found that at both global and regional scales, microbial community assembly partitioned by trophic mode. Globally, taxa with a phototrophic lifestyle, encompassing both oxygenic and anoxygenic phototrophy, were highly exclusive to specific climate regimes, while taxa with a chemoheterotrophic lifestyle had a more cosmopolitan distribution. Regional inputs such as substrate properties and local moisture gradients also affected *Cyanobacteria* more than other phyla. We argue these results demonstrate differential selective pressures between producers and consumers that drive endolithic community assembly.

Producers in the endolithic community are likely more sensitive to climatic conditions such as water availability, solar irradiance, and temperature due to their photosynthetic requirement and status as pioneering organisms in endolithic communities (Tiano et al., 1995; Crispim and Gaylarde, 2005). In hyper-arid and hot deserts, *Chroococcidiopsis* is the only oxygenic photosynthesizer found in the community; this has been attributed to the superlative desiccation and radiation resistance properties of the taxon (Billi et al., 2000, 2011). It has been argued that the combination of hot and hyper-arid conditions constitutes the most extreme terrestrial environment on Earth (Friedmann, 1980; Wierzchos et al., 2018), suggesting that the absence of other types of phototrophs in the most extreme deserts is likely the result of stringent environmental filtering. As the electron donor for oxygenic photosynthesis, liquid water is likely a powerful selector for producers in the endolithic community, and indeed even a modest increase in precipitation within the Namib Desert resulted in a large increase in the diversity of *Cyanobacteria*. Interestingly, this effect was also seen among anoxygenic phototrophic taxa, which were highly specific to higher moisture regimes. Producers in polar communities were dominated by eukaryotic *Trebouxia* while *Cyanobacteria* were only sparsely present, suggesting that prokaryotic and eukaryotic producers compete for access to the limited space within the rock suitable for photosynthesis. This is supported by the high number of secondary metabolites, especially antimicrobial compounds, that are found in endolithic *Cyanobacteria* (Crits-Christoph et al., 2016). *Trebouxia*, as well as the fungal classes *Lecanoromycetes* and *Eurotiomycetes* are well-recognized for their tendency to form lichen symbionts (Ahmadjian, 1988; Miadlikowska et al., 2006; Geiser et al., 2008), which are particularly well adapted to the polar environments because they are more cold-resistant than the photobiont or mycobiont components alone (Barták et al., 2007). Physiological studies also show that endolithic lichens begin to photosynthesize at lower water potentials than *Chroococcidiopsis*, allowing them to better utilize the abundant relative humidity in polar deserts (Palmer and Friedmann, 1990). High-resolution sequencing of the semi-arid Escalante site revealed a scale of diversity previously undescribed in desert endolithic systems, with a community supporting robust prokaryotic and eukaryotic phototrophic populations. It has been suggested that local geochemistry, in particular pH, plays a more important role in driving community structure under these conditions (Walker and Pace, 2007).

Microbial consumers exhibited higher stochasticity across deserts at both a global and regional scale. Examination of *Proteobacteria* and *Actinobacteria* across the Namib Desert moisture gradient found little difference in diversity, suggesting that consumer assembly is less sensitive to climate-based selection than producer assembly. Features of some consumer taxa, such as desiccation resistance and spore formation may also promote their long-range dispersal in the atmosphere and contribute to the more cosmopolitan global distribution we observed (Archer et al., 2019). Despite this, network analysis showed that chemoheterotrophic taxa still tended to cluster according to their respective climate regime, reinforcing the view that consumers still remain organized according to environmental influences. However, the patterns of consumer assembly we observed were more difficult to explain than those of producer assembly. One likely explanation is that biotic influences play a significant role in driving consumer profiles across deserts, including specific interactions with producers and predation from viruses (Rodriguez-Brito et al., 2010; Valverde et al., 2015; Fernández et al., 2018), but these dynamics are poorly studied in the endolithic system.

The partitioning of assembly influences by trophic level has been proposed before in studies of the hypolithic system but these studies do not concur on how producer and consumer assemblies are different (Caruso et al., 2011; Lacap-Bugler et al., 2017). We also note that these previous studies reported modest assembly according to environmental niche and the reported effects were small when compared to the strong climate-based clustering we observed within the endolithic system. Hypolithic communities are contiguous with the underlying soil and recruited in part from local soil populations (Makhalanyane et al., 2013), which may explain the increased stochasticity observed in hypolithic community assembly. In contrast, endolithic communities are completely enclosed within the rock environment and are further influenced by unique properties of the rock substrate, such as geochemistry, water retention, and light attenuation (Meslier et al., 2018). We suggest that complex assembly processes may be more discernible in the endolithic system because the assembly of these communities is influenced by more tractable environmental variables; thus the endolithic system may prove promising for further investigations of microbial biogeography.

## Conclusion

This study characterized for the first time global patterns of endolithic community diversity using high-throughput sequencing. We found that climate-based environmental selection was the primary organizer of endolithic community composition. This study joins several others in demonstrating that desert microbiomes are highly sensitive to climatic conditions, highlighting a need for studies of these fragile communities in the context of changing climate. The importance of rock substrate properties in the endolithic community is also evident, and we found a unique chasmoendolithic sandstone community in the Namib Desert, which demonstrates that within a single desert, substrate effects may be equally or more important than microclimate in organizing community composition. This study also reveals that phototrophs and heterotrophs within the endolithic community organize according to different assembly influences, with phototrophs being more sensitive to climate and substrate effects than heterotrophs. These findings emphasize that the functional niche is an essential consideration in studies of microbial assembly, and future research on lithic systems should consider using meta-omic methods to investigate community function along with taxonomy.

## Data Availability Statement

Raw sequencing data are available from the National Center for Biotechnology Information Sequence Read Archive (SRA) under BioProject ID PRJNA561762.

## Author Contributions

EQ contributed to the design of the study, conducted the experiments, analyzed the data, and wrote the manuscript. JD, AO, and DC collected the field samples. GM-K provided support for fieldwork in the Namib Desert. CO collected field samples and took images from the Canadian Arctic. VM and JD conceived and contributed to the design of the study. JD supervised the project and edited the manuscript. All authors approved the final version for submission.

## Conflict of Interest

The authors declare that the research was conducted in the absence of any commercial or financial relationships that could be construed as a potential conflict of interest.
