# Supplementary material for: Trophic Selective Pressures Organize the Composition of Endolithic Microbial Communities From Global Deserts
Source: Front Microbiol. 2020 Jan 8;10:2952. doi: 10.3389/fmicb.2019.02952 (PMC6960110; doi:10.3389/fmicb.2019.02952)
Supplement: Supplementary file 1 [file Data_Sheet_1.pdf]

Trophic Selective Pressures Organize the Composition of Endolithic Microbial Communities from Global Deserts
Supplementary Materials

Evan B. Qu[1], Chris C. Omelon[2], Aharon Oren[3], Victoria Meslier[1], Don A. Cowan[4], Gillian Maggs-Kölling[5], and Jocelyne DiRuggiero[1§]

[1]*Johns Hopkins University, Department of Biology, Baltimore, USA*
[2] *Queen's University, Department of Geography and Planning, Kingston, Ontario, Canada* [3]*The Hebrew University of Jerusalem, Institute of Life Sciences, Edmond J. Safra Campus, Jerusalem, Israel*
[4]*Centre for Microbial Ecology and Genomics, Department of Biochemistry, Genetics and Microbiology, University of Pretoria, Pretoria, South Africa*
[5]*Gobabeb-Namib Research Institute, Walvis Bay, Namibia*

Table of Contents

Table S1: Sampling sites and dates, number of rocks sequenced per site
Table S2: Weather data from sites.
Table S3: Chemical composition of sandstone samples
Table S4: Water-soluble ions from sandstone samples
Table S5: Sandstone physical properties
Table S6: Alpha diversity by site
Table S7: Per-site relative abundances of potential anoxygenic phototrophic clades, based on partial 16S rRNA sequences

Figure S1: Diversity rarefaction curves
Figure S2: Grain size distributions for sandstones
Figure S3: Venn diagrams showing distribution of bacterial OTUs
Figure S4: Heatmap of Namib Desert Actinobacteria

Scripts used for analyses of sequence data

Desert	Site Name	GPS coordinates	Collection Date	No. 16S rRNA samples	No. ITS samples
Negev Desert, Israel	Ramon Crater	30°37'20"N, 34°50'38"E	Sep-2016	10	-
	Timna Park	29°47'25"N, 34°58'2"E	Sep-2016	10	-
Namib Desert, Namibia	Namib Coastal	23°32'12"S, 14°59'36"E	Apr-2017	10	-
	Namib Central	23°40'32"S, 15°16'24"E	Apr-2017	10	-
	Namib Far East	23°45'22"S, 15°46'14"E	Apr-2017	10	-
Colorado Plateau, USA	Escalante	37°45'5"N, 111°26'32"W	Jul-2018	10	6
Canadian Arctic	Cape Bounty	74˚54'26"N 109˚35'46"W	May-2018	9	10
	Eureka	79°59'21"N, 85°56'02"W	May-2018	10	2
McMurdo Dry Valleys, Antarctica	University Valley	77°51'52"S, 160°43'31"E	Jan-2013	10	7

Table S1: Sampling sites and dates, number of rocks sequenced per site.

Desert		Negev Desert, Israel[1,2]		Namib Desert, Namibia[3]			Colorado Plateau, USA[6,7]	Canadian Arctic[4,5]		McMurdo Dry Valleys, Antarctica[8,9]
**Sampling site**		Ramon Crater	Timna Park	Coastal	Central	Far East	Escalante	Eureka	Cape Bounty	University Valley
**Elevation (m.a.s.l.)**		865	255	386	586	1030	1726	10	88	1700
**Air Temperature (°C)**	**Ave**	18.6	26.8	21.4	21.9	22.7	12.3	-17.6	-14.3	-23.4
	**Min**	0.7	6.3	-0.6	8.7	8.6	-6.8	-50.5	-49.4	-45.0
	**Max**	39.7	45.7	43.3	32.9	32.7	34.7	18.5	17.4	5.0
**RH (%)**	**Ave**	53	36	48	41	30	82	77	91	46
	**Min**	3	5	25	9	5	25	27	32	10
	**Max**	100	90	64	87	79	100	100	100	99
**Precipitation (mm/yr)**		37[a]	16[a]	14	36	62	232	48	57	0[b]
**Daily solar radiation (W/m^2/day)**		223		275			225	92		103

[1]Israel Meteorological Service - Mitzpe Ramon and Eilat stations; <http://www.ims.gov.il> **|** 2015-7 to 2017-7
[2]Evseev and Kudish, Analysis of solar irradiation measurements at Beer Sheva, Israel from 1985 through 2013, *Energy Conservation and Management*. 97: 307-314 (2015) **|** 2012-1 to 2013-8
[3]Sasscal WeatherNet - Gobabeb Met, Garnet Koppie, Ganab stations; <http://www.sasscalweathernet.org> **|** 2015-7 to 2017-7
[4]Environment and Natural Resources, Government of Canada **|** 2016-1 to 2017-12
[5]Cape Bounty Arctic Watershed Observatory; <https://capebountyresearch.com/> **|** 2016-1 to 2017-12
[6]NOAA National Centers for Environmental Information - Escalante and Bryce Canyon stations; <https://www.ncdc.noaa.gov> **|** 2017-1 to 2018-12
[7]NOAA National Solar Radiation Database - Bryce Canyon Airport station; <https://www.ncdc.noaa.gov> **|** 2010-1 to 2010-12
[8]Lacelle et al, Solar Radiation and Air and Ground Temperature Relations in the Cold and Hyper-Arid Quartermain Mountains, McMurdo Dry Valleys of Antarctica, *Permafrost and Periglac. Process*. 27: 163–176 (2016) **|** 2010-1 to 2012-1
[9]McMurdo Dry Valleys LTER - Beacon valley station; <https://www.mcmlter.org/> **|** 2010-1 to 2012-12

[a]Rainfall data were taken from 2017-2018 due to abnormal rainfall events after sample collection in 2016
[b]University Valley receives no liquid rainfall, all moisture is from humidity and meltwater

Table S2: Weather data from sites. Average, minimum and maximum weather data for a two-year period encompassing the sampling date of each site. Data sources and special notes are listed below the table.

%	Namib Desert			Negev Desert		Colorado Plateau	Canadian Arctic		McMurdo Dry Valleys
	Coastal	Central	Far East	Ramon Crater	Timna Park	Escalante	Cape Bounty	Eureka	University Valley
**SiO_2**	83.9	60.7	81.1	91.0	88.6	92.7	92.7	98.5	96.3
**Al_2O_3**	4.45	3.60	3.79	0.82	3.07	3.24	2.86	0.30	0.39
**Fe_2O_3**	3.60	3.79	4.80	8.40	7.01	0.82	2.09	1.08	3.78
**MgO**	1.55	0.75	1.14	0.03	0.05	0.16	0.14	0.05	ND
**CaO**	0.47	15.8	1.28	0.53	0.08	0.07	0.08	0.17	0.02
**K_2O**	1.59	1.14	1.67	0.08	1.69	1.57	1.69	0.06	0.05
**Na_2O**	0.57	0.68	0.87	0.13	0.10	0.09	0.10	0.06	0.07
**TiO_2**	0.72	0.30	0.44	0.19	0.09	0.09	0.08	0.08	0.02
**MnO**	0.06	0.06	0.07	0.08	0.07	0.01	0.02	0.01	0.03
**P_2O_5**	0.03	0.03	0.04	0.02	0.02	0.02	0.02	0.01	ND
**Cr_2O_3**	0.01	ND	0.02	ND	ND	0.06	ND	0.11	ND
**V_2O_5**	0.01	ND	ND	ND	ND	ND	ND	ND	ND
**Loss on Ignition**	2.93	13.5	3.06	0	0	0.971	0.392	0.616	0
**Sum**	99.9	100.4	98.3	101.3	100.8	99.8	100.2	101.0	100.7

Table S3: Chemical composition of sandstones. Sandstone chemical composition was measured with X-ray fluorescence mass spectrometry. Loss on Ignition represents the percent of sandstone that was made of volatile substances. ND = below detection limit.

**All units in mg/kg**	**Negev Desert**		**Namib Desert**			**Colorado Plateau**	**Canadian Arctic**		**McMurdo Dry Valleys**
	Ramon Crater	Timna Park	Coastal	Central	Far East	Escalante	Cape Bounty	Eureka	University Valley
**Anions**									
NO_3^-, as N	0.3	1.76	2.32	3.26	0.94	1.12	0.24	0.26	0.2
PO_4^{3-}, as P	ND	ND	ND	ND	ND	ND	ND	0.2	ND
Cl^-	2	14	78	16	2	4	20	88	2
SO_4^{2-}	6	30	20	18	2	ND	14	4	4
**Cations**									
Ca^{2+}	24	28	12	16	18	2	30	10	6
Mg^{2+}	ND	ND	6	4	4	ND	16	6	ND
Na^+	2	8	62	22	4	2	12	58	4
K^+	4	16	16	20	20	6	10	10	2
**Dissolved Metals**									
Fe^{2+}, Fe^{3+}	ND	ND	ND	ND	ND	ND	ND	ND	ND

Table S4: Water-soluble ions from sandstones. Water soluble ions were extracted from crushed sandstone and measured with ion chromatography and inductively coupled plasma atomic emission spectroscopy. Units represent mg of ions per kg of rock. ND = below detection limit.

	D50 Grain size (µm)	Percent water retention
Ramon Crater	268	23.2 ± 1.7
Timna Park	530	14.9 ± 0.4
Namib Coastal	308	27.0 ± 1.3
Namib Central	233	11.0 ± 2.9
Namib Far East	172	30.3 ± 15.2
Escalante	150	11.8 ± 7.0
Cape Bounty	239	12.1 ± 2.1
Eureka	116	18.2 ± 1.1
University Valley	282	10.9 ± 0.7

Table S5: Sandstone physical properties. 50th percentile diameter (D50) of grain size distribution and percent water retention measured with a water resaturation method.

	Prokaryotic Diversity		Eukaryotic Diversity	
	OTUs	Shannon	ESVs	Shannon
Ramon Crater	41 ± 5	4.57 ± 0.24	-	-
Timna Park	54 ± 7	5.11 ± 0.19	-	-
Namib Coastal	50 ± 8	4.57 ± 0.28	-	-
Namib Central	71 ± 4	5.61 ± 0.11	-	-
Namib Far East	92 ± 4	6.03 ± 0.07	-	-
Escalante	252 ± 14	7.51 ± 0.10	60 ± 13	3.28 ± 0.48
Cape Bounty	105 ± 17	6.03 ± 0.25	74 ± 14	4.46 ± 0.37
Eureka	125 ± 7	6.57 ± 0.09	29 ± 10	3.68 ± 0.27
University Valley	31 ± 5	4.37 ± 0.20	20 ± 2	3.26 ± 0.18

Table S6: Alpha diversity by site. Mean and standard deviation of observed OTUs/ESVs and Shannon index at each site, based on 16S rRNA gene sequences for prokaryotes and ITS sequences for eukaryotes.

	Alphaproteobacteria			*Betaproteobacteria*		*Gemmatimonadetes*
	Acetobacteraceae	*Rhodospirillaceae*	*Rhodobacteraceae*	*Bradyrhizobiaceae*	*Comamonadaceae*	*Gemmatimonas*
**University Valley**	0.00%	0.00%	0.00%	0.00%	0.00%	0.00%
**Cape Bounty**	0.35%	0.00%	0.20%	0.31%	0.24%	0.02%
**Eureka**	0.00%	0.02%	2.52%	0.00%	0.00%	0.00%
**Escalante**	0.00%	0.13%	0.94%	0.04%	0.87%	0.29%
**Namib Coastal**	0.00%	0.00%	0.14%	0.00%	0.17%	0.14%
**Namib Central**	0.00%	0.31%	0.74%	0.00%	0.05%	0.32%
**Namib Far East**	0.00%	0.02%	0.76%	0.00%	0.87%	0.07%
**Ramon Crater**	0.00%	0.00%	0.66%	0.00%	0.00%	0.00%
**Timna Park**	0.00%	0.00%	0.03%	0.00%	0.21%	0.00%

Table S7: Per-site relative abundances of putative anoxygenic phototrophic clades based on 16S rRNA amplicon sequences.

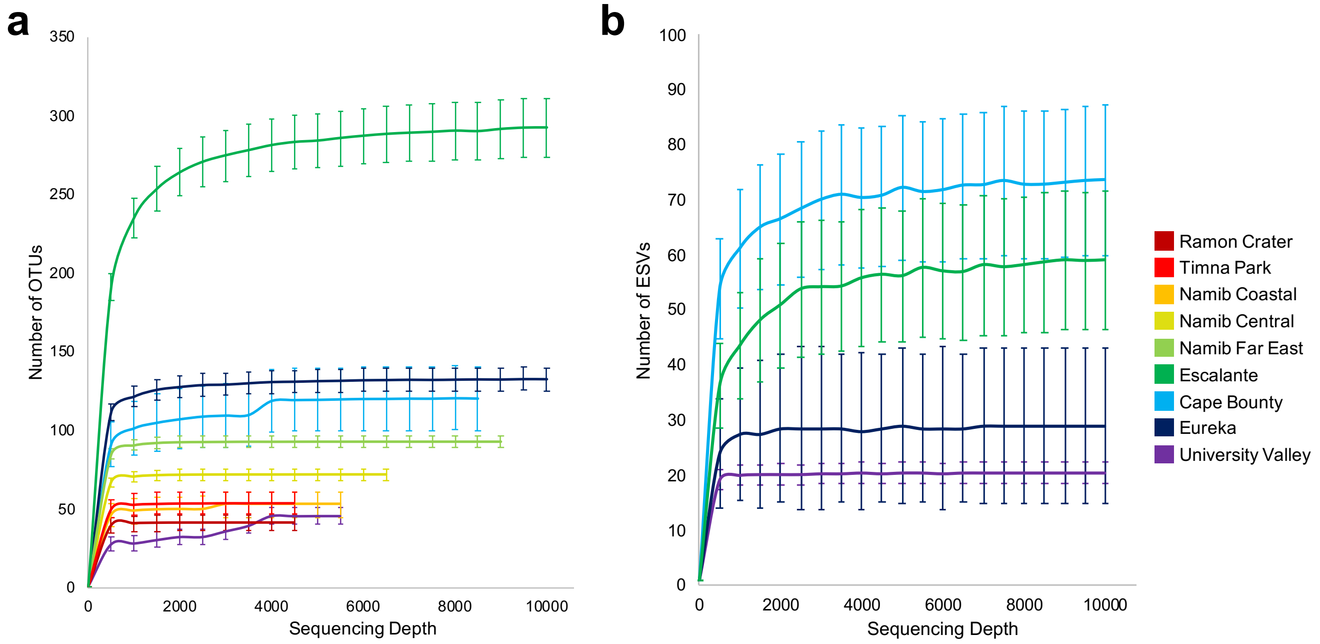

Figure S1: Diversity rarefaction curves. Rarefaction curves for **(a)** prokaryotic OTUs and **(b)** eukaryotic ESVs at 500-sequence sampling intervals, up to 10,000 sequences. These curves show that diversity approaches asymptote before maximum sequencing depth is reached

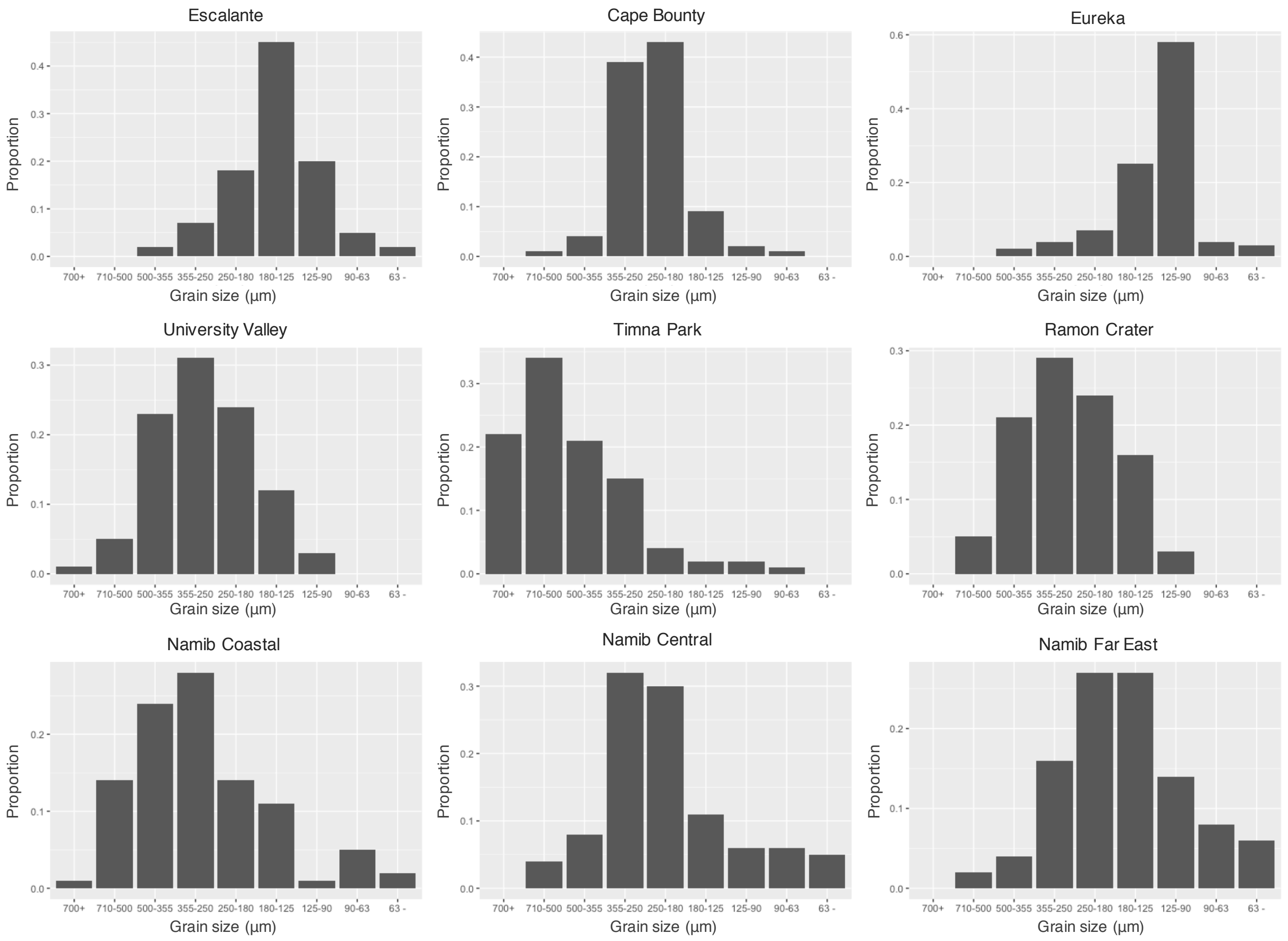

Figure S2: Grain size distributions for sandstones. Histograms showing grain size distributions for sandstones at each site.

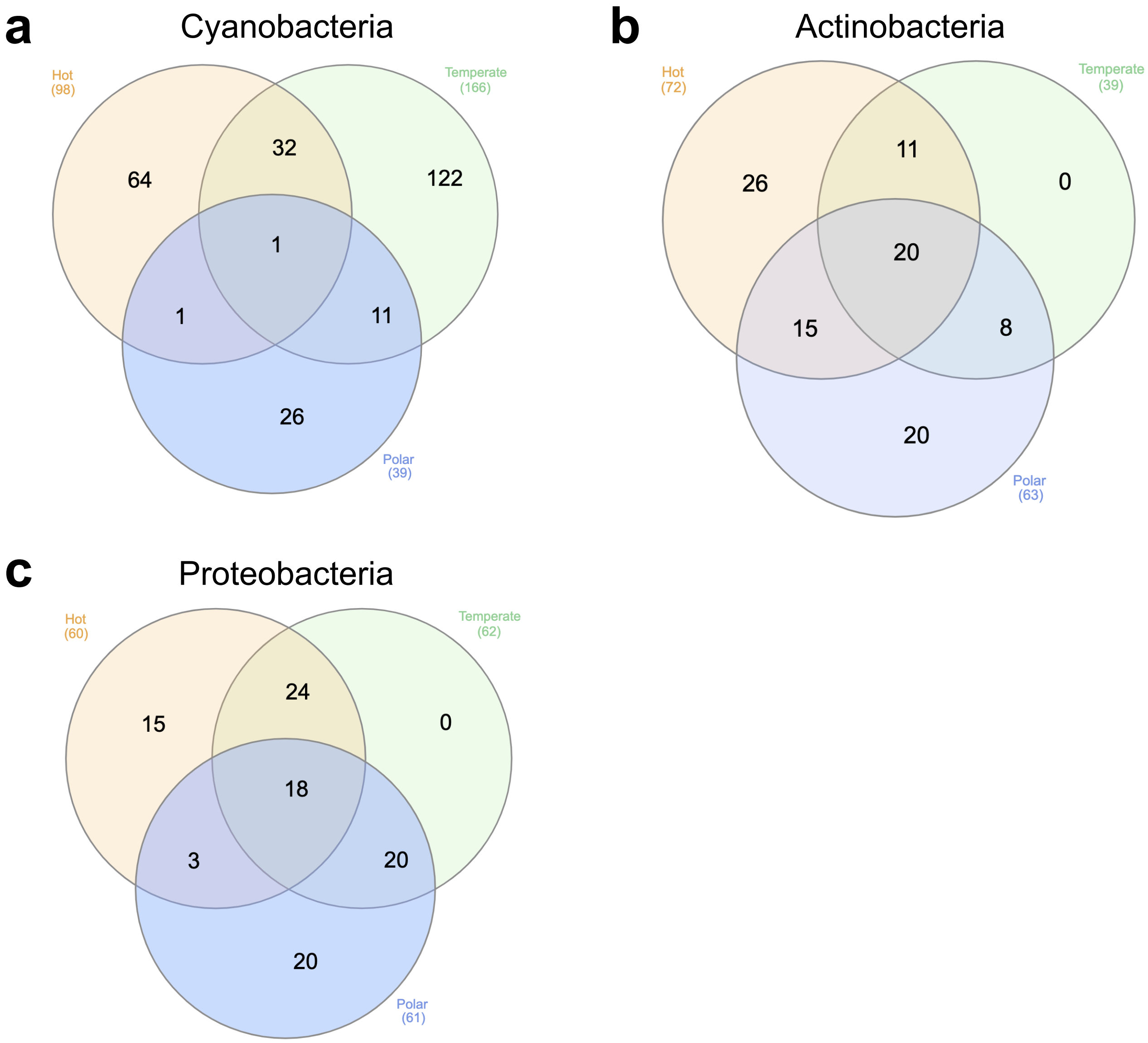

Figure S3: Venn diagram showing distribution of **(a)** all Cyanobacteria OTUs, **(b)** 100 most abundant Actinobacteria OTUs, and **(c)** 100 most abundant Proteobacteria OTUs between the three climate regimes (hot, polar, and temperate).

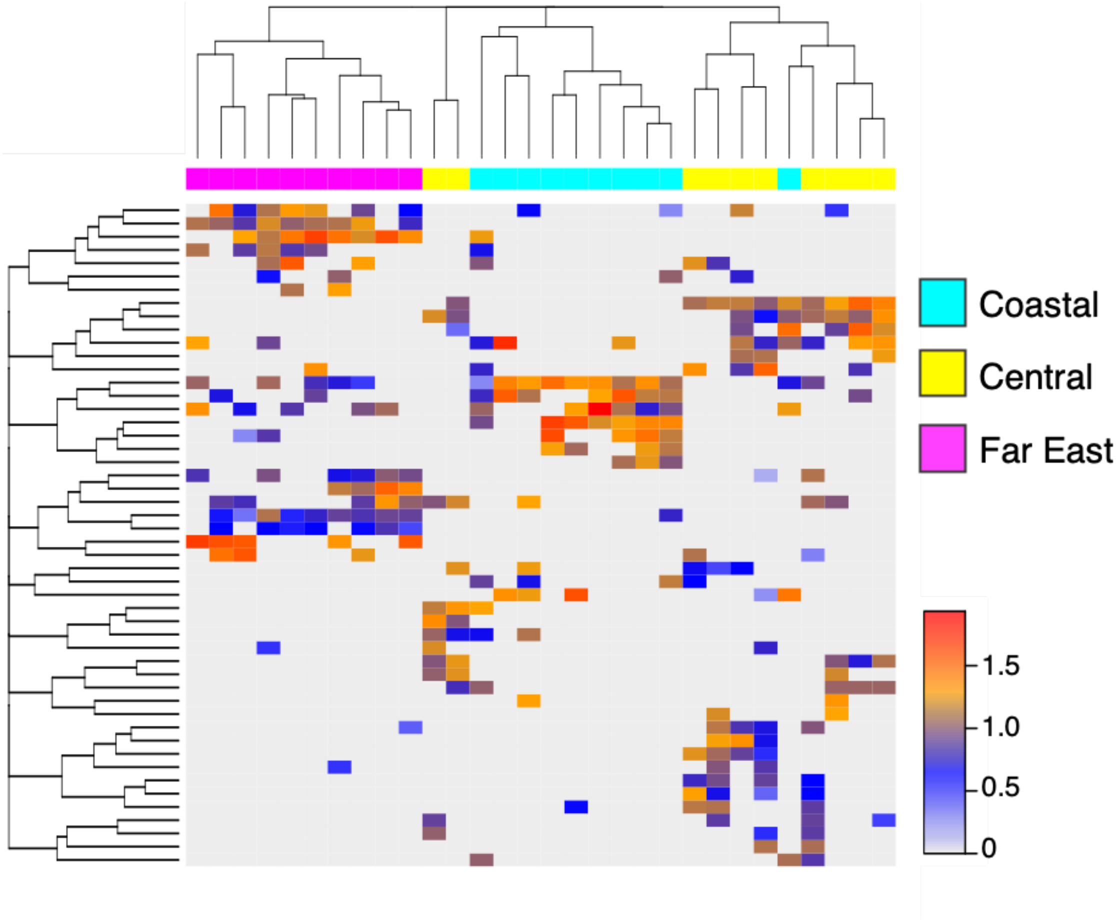

Figure S4: Heatmap of Namib Desert Actinobacteria. Actinobacteria OTUs from three sites in the Namib Desert. Rows correspond to OTUs and columns correspond to individual samples. Color scale represents log-normalized relative abundances.

Scripts used for analyses of sequence data

```
## 16s analysis pipeline using QIIME2 and sequence variant picking
with DADA2

source activate qiime2

## step 1: import your sequences into a QIIME artifact using a
manifest file
# manifest file is a .csv file that gives the fwd and rev filenames
for paired end reads
     # 3 columns:
     # (1)'sample-id': name of sample
     # (2)'absolute-filepath': path to file
     # (3)'direction': 'forward' or 'reverse'
#'sed 's/"//g' < infile > outfile' to remove quotes if you make in
Excel

qiime tools import \
     --type SampleData[PairedEndSequencesWithQuality] \
     --input-path manifest.csv \
     --output-path sequences.qza \
     --input-format PairedEndFastqManifestPhred33 \

# view read count and phred scores for your data
qiime demux summarize --i-data sequences.qza --o-visualization
sequences.qzv

## step 2: using dada2 package within QIIME2 for denoising, merging,
and variant picking
# adjust trunc/trim lengths as needed depending on your quality plot

qiime dada2 denoise-paired \
     --i-demultiplexed-seqs sequences.qza \
     --p-trunc-len-f 250 \
     --p-trunc-len-r 250 \
     --p-trim-left-f 0 \
     --p-trim-left-r 0 \
     --output-dir dada2-out \
     --verbose \
```

```
# cluster sequences open reference at 0.97 identity with vsearch
package
# use the latest edition of SILVA
qiime vsearch cluster-features-open-reference \
     --i-table dada2-out/table.qza \
     --i-data dada2-out/rep-seqs.qza \
     --i-reference-sequences 97_silva.qza \
     --p-perc-identity 0.97 \
     --p-strand both \
     --output-dir 97-cluster \
     --verbose \

# visualize feature table and rep seqs
qiime feature-table summarize \
     --i-table feature-table.qza \
     --o-visualization feature-table.qzv \
     --m-sample-metadata-file metadata.txt

qiime feature-table tabulate-seqs \
     --i-data rep-seqs.qza --o-visualization rep-seqs.qzv

## step 3: cluster with vsearch into 97% identity OTUs
## for reference sequences, use SILVA 16S database at 0.97 identity

qiime vsearch cluster-features-open-reference \
     --i-sequences rep-seqs.qza \
     --i-table feature-table.qza \
     --i-reference-sequences \
     --p-perc-identity 0.97 \
     --p-strand both \
     --output-dir 97-clust/

## step 4: assign taxonomy (requires trained classifier)
qiime feature-classifier classify-sklearn \
     --i-classifier classifier.qza \
     --i-reads repseqs.qza \
     --o-classification taxonomy.qza

# visualize taxonomy barplots
qiime taxa barplot \
--i-table otu-table.qza \
```

```
--i-taxonomy taxonomy.qza \
--m-metadata-file metadata.txt \
--o-visualization taxa-bar-plots.qzv


## step 5: make rooted tree of ref-seqs

qiime alignment mafft   --i-sequences rep-seqs.qza   --o-alignment
aligned-rep-seqs.qza
qiime alignment mask   --i-alignment aligned-rep-seqs.qza   --o-
masked-alignment masked-rep-seqs.qza
qiime phylogeny fasttree   --i-alignment masked-rep-seqs.qza   --o-
tree unrooted-tree.qza
qiime phylogeny midpoint-root   --i-tree unrooted-tree.qza   --o-
rooted-tree rooted-tree.qza

## step 6: run core alpha and beta diversity metrics
# adjust sampling depth as needed
qiime diversity core-metrics \
     --i-phylogeny rooted-tree.qza \
     --i-table feature-table.qza \
     --p-sampling-depth $DEPTH \
     --m-metadata-file metadata.txt
     --output-dir core-metrics-results

##step 7: statistical tests
# adonis test
qiime diversity beta-group-significance \
     --i-distance-matrix weighted_unifrac_distance_matrix.qza \
     --m-metadata-file metadata.txt \
     --p-method permanova \
     --metadata-column XXX \
     --o-visualization adonis.qzv

#mantel test
#first, create a euclidean distance matrix from your metadata
qiime metadata distance-matrix \
     --i-metadata-file metadata.txt \
     --m-metadata-column XXX \
     --o-distance-matrix metadata_distance_matrix.qza
```

```
qiime diversity mantel \
     --i-dm1 weighted_unifrac_distance_matrix.qza \
     --i-dm2 metadata_distance_matrix.qza \
     --p-method pearson \
     --p-intersect-ids True \
     --o-visualization mantel.qzv

#pearson test for alpha diversity
qiime diversity alpha-group-significance \
     --i-alpha-diversity alpha.qza \
     --m-metadata-file metadata.txt \
     --o-visualization pearson.qzv


## step 8: making bipartite networks in QIIME1
source activate qiime1
make_otu_network.py -i actino-table-wtax.biom -m actino-mapping.txt -
o actino-network
#export to cytoscape
```
